# Robust Machine Learning for Colorectal Cancer Risk Prediction and Stratification

**DOI:** 10.3389/fdata.2020.00006

**Published:** 2020-03-10

**Authors:** Bradley J. Nartowt, Gregory R. Hart, Wazir Muhammad, Ying Liang, Gigi F. Stark, Jun Deng

**Affiliations:** ^1^Department of Therapeutic Radiology, Yale University, New Haven, CT, United States; ^2^Department of Radiation Oncology, Medial College of Wisconsin, Milwaukee, WI, United States; ^3^Department of Statistics & Data Science, Yale University, New Haven, CT, United States

**Keywords:** colorectal cancer, risk stratification, neural network, concordance, self-reportable health data, external validation

## Abstract

While colorectal cancer (CRC) is third in prevalence and mortality among cancers in the United States, there is no effective method to screen the general public for CRC risk. In this study, to identify an effective mass screening method for CRC risk, we evaluated seven supervised machine learning algorithms: linear discriminant analysis, support vector machine, naive Bayes, decision tree, random forest, logistic regression, and artificial neural network. Models were trained and cross-tested with the National Health Interview Survey (NHIS) and the Prostate, Lung, Colorectal, Ovarian Cancer Screening (PLCO) datasets. Six imputation methods were used to handle missing data: mean, Gaussian, Lorentzian, one-hot encoding, Gaussian expectation-maximization, and listwise deletion. Among all of the model configurations and imputation method combinations, the artificial neural network with expectation-maximization imputation emerged as the best, having a concordance of 0.70 ± 0.02, sensitivity of 0.63 ± 0.06, and specificity of 0.82 ± 0.04. In stratifying CRC risk in the NHIS and PLCO datasets, only 2% of negative cases were misclassified as high risk and 6% of positive cases were misclassified as low risk. In modeling the CRC-free probability with Kaplan-Meier estimators, low-, medium-, and high CRC-risk groups have statistically-significant separation. Our results indicated that the trained artificial neural network can be used as an effective screening tool for early intervention and prevention of CRC in large populations.

## Introduction

Of all new cancer incidences in the United States, 8.1% are colorectal cancer (CRC) (Falco et al., 2018; National Cancer Institute, 2018). The 5-year survival rate for CRC ranges from 14% for a distant stage to 90% for a localized stage. CRC is responsible for 8.3% of all cancer deaths, and is especially deadly and recurrent when coincident with diabetes and hypertension (Yang et al., 2012). However, there exists little knowledge of the primary causes of CRC. Thus, current screening recommendations are only based on family history of CRC and age. Specifically, the United States Preventative Services Task Force (USPSTF) recommends screening for individuals between ages 50 and 75 while the American Cancer Society recommends screening for individuals between ages 45 and 75 (Collins et al., 2015; Bibbins-Domingo et al., 2016). Both guidelines recommend screening for anyone with one or more primary relatives who have ever had CRC. While screening according to these guidelines indisputably saves lives, high-risk individuals with no CRC family history and/or aged 18–49 would clearly benefit from a model that better detects their risk. Low-risk individuals that are flagged for screening under a new model (Collins et al., 2015; Bibbins-Domingo et al., 2016), would also be given information to help them choose whether they want to be subject to invasive, expensive, and injurious (Benard et al., 2018; National Cancer Institute, 2019) screening. Hence, it is important to develop an effective method to estimate CRC risk non-invasively and cost-effectively.

There have been a lot of previously-developed CRC-risk models that do not involve biomarkers (Usher-Smith et al., 2016). Using only professionally-collected routine data (biological sex, use of non-steroidal anti-inflammatory drugs (NSAIDs), form of recruitment, non-specific abdominal pain, bowel-habit, age, BMI, cholesterol, and triglycerides), Betes et al. achieved a concordance ~0.7 using a multiple logistic regression model (Betes et al., 2003). Using data from a self-completed questionnaire (asking about CRC in first-degree relatives, BMI, screening, NSAID use, diet, inflammatory bowel disease, alcohol/tobacco use, and physical activity), Colditz et al. also built a multiple logistic regression model of similar concordance ~0.7 from data on family history, obesity, screening, diet (multivitamin, alcohol, vegetables, and red meat consumption), height, physical activity, pharmaceuticals (prophylactic, post-menopausal hormone, and aspirin use), and inflammatory bowel disease (Colditz et al., 2000). Both models are externally tested, i.e., the model is built from one dataset and its performance is reported on a dataset from a separate study (Collins et al., 2015). However, compared to the simple logistic regression models, there has been no systematic study on the development of more advanced machine learning models for CRC risk prediction and stratification for a large population, in consideration of various imputation methods.

Hence in this work, we aim to identify an effective mass screening method for CRC risk based solely on personal health data. We trained and cross-tested various machine learning models with two large national databases, reporting performance in terms of the concordance, a performance metric that is biased but standard (Hanley and McNeil, 1982; Hosmer and Lemeshow, 2000; Fawcett, 2005; Hajian-Tilaki, 2013). A variety of imputation methods were explored in handling the missing data. Additionally, a component of cross-uncertainty is incorporated to the total uncertainty reported, adding stringency to our testing that to our knowledge has not been used before. Finally, we furnish some ideas on how our model can be deployed for real world applications.

## Materials and Methods

### Two Datasets From Separate Studies

The National Health Interview Survey (NHIS) dataset^1^ is a cross-sectional study of the overall health status of the United States. Each year, roughly 30,000 adults are interviewed on a range of current and past personal health conditions. The first survey of the NHIS after a significant revision was administered in 1997 and the next such redesign of the NHIS is scheduled to appear in 2019, so data from years 1997 to 2017 was used. Our other study is the longitudinal Pancreatic, Lung, Colorectal, Ovarian (PLCO) Cancer Screening dataset from the National Cancer Institute^2^. The PLCO dataset is a randomized, controlled longitudinal study on the efficacy of screening for prostate, lung, colorectal, and ovarian cancer. Between November 1993 and July 2001, participants were randomized, entered into the trial, answered a baseline questionnaire (BQ), and were followed for up to 14 years, exiting the trial early if they were diagnosed with any cancer or if they died. To match this PLCO data with the NHIS dataset, we assumed that answering the PLCO BQ was equivalent to participating in the NHIS's interview.

Data was marked by 7 for responses of “Refused,” 8 for “Not ascertained,” and 9 “Don't know” in the NHIS^1^; all these responses were assumed to indicate data missing completely at random (Little and Rubin, 2014) (MCAR). This is distinguished from data not missing at random, which is marked by the table entry being actually blank (e.g., all pregnancy data has a blank entry for male respondents). PLCO uses the same scheme of marking the missingness of data with digit-entries, while data missing not at random is actually blank.

The United States Preventative Services Task Force guidelines currently recommend anyone with family history of CRC and/or aged 50–75 years for screening (Bibbins-Domingo et al., 2016), while screening at ages 76+ is up to the individual. Thus, ages 18–49 and ages 50–75 form sub-demographics of data that are of interest. To assess performance in these sub-demographics, we trained and tested models on these age splits of the data as well as on all ages.

There are factors appearing in the NHIS dataset but missing in the PLCO dataset, and vice versa. Specifically, factors appearing in the NHIS but not in the PLCO are alcohol-use, vigorous exercise frequency, functional limitations, kidney comorbidity, and incidence of angina. Factors appearing in the PLCO but not in the NHIS are non-steroidal anti-inflammatory drug (NSAID) use, gallbladder inflammation, and incidence of diverticulitis. To ensure a TRIPOD level 3 cross-testing between separate datasets, and all its rigor and robustness, all these factors are not used in our study.

### CRC vs. Never Cancer

The NHIS records each respondent's age at the time of the survey, and the age(s) at which the respondent was diagnosed with cancer of the colon and/or rectum, if at all. Respondents were counted as positive cases of CRC if their diagnosis happened <4 years prior to the survey. In each study, a small fraction of the respondents were recently diagnosed with CRC. We considered CRC in survey respondents ages 18–85.

In PLCO and NHIS, the following four types of respondents were discarded: (1) those diagnosed with CRC more than 4 years prior to taking the survey (NHIS) or answering the questionnaire (PLCO), (2) those non-CRC respondents diagnosed with any other cancer at any time, (3) those CRC respondents diagnosed with a cancer other than CRC at a time before their CRC diagnosis, and (4) those CRC respondents having CRC at a time before randomization (PLCO only). Members of the first group were discarded because their reported personal health data was considered irrelevant to their CRC diagnosis. Those in the second and third groups were discarded because those diagnosed with any cancer already receive heightened screening attention, defeating the purpose of assessing their risk. The fourth group is discarded because before randomization the time (in days) of CRC diagnosis is not known. Thus, the negative examples were those who were never diagnosed with any cancer and are referred to as “never-cancer” (NC) while the positive examples were those recently diagnosed with CRC and are referred to as “CRC.”

To be considered a positive case of CRC in the PLCO data remaining after the deletion described above, the respondent needed to meet both of the following conditions: (1) they were diagnosed with CRC within 4 years of the BQ and (2) CRC was the first cancer they had. If both of these conditions were not met, the respondent was considered part of the non-cancer population remaining after the above discarding was carried out. Hence, the outcome variable used in both datasets was the respondent's cancer status coded to a 0 or a 1. A value of 0 indicated that the respondent was never diagnosed with cancer (CRC or any other cancer). It is assumed that a given respondent would have already been flagged for screening if previously diagnosed with any kind of cancer, defeating the purpose of risk-scoring. A value of 1 indicated that the respondent was diagnosed with CRC within four (4) years of answering either the PLCO BQ or the NHIS interview questions. All respondents who fit neither of these criteria were assumed to be data not missing at random (Little and Rubin, 2014), and thus discarded (never subject to any imputation methods).

Performance after training with such an outcome variable is not relative to the sensitivity and specificity of any gold standard. In our work, the gold standard of CRC diagnosis is colonoscopy. Unfortunately, colonoscopy data is missing not at random for a significant portion of data. Specifically, only NHIS questionnaires from years 2000, 2005, 2010, and 2015 asked the respondent if they had ever been screened by the gold standard (sigmoidoscopy, colonoscopy, or proctoscopy). We therefore assumed that neither dataset contained any false positive or false negative cases.

### Data Preparation

For reproducibility, we describe how the raw data was mapped to the datasets used to train and test the machine learning algorithms (MLAs). The factors of ever having hypertension, ulcers, a stroke, any liver comorbidity, arthritis, bronchitis, coronary heart disease, myocardial infarction, and/or emphysema are binary variables and mapped to 0 for “no” and 1 for “yes.” Diabetic status has one of three discrete values: not diabetic, pre-diabetic/borderline, and diabetic, respectively. These conditions were mapped to 0, 0.5, and 1, respectively. The age factor is continuous and equals the age at response to the NHIS or PLCO BQ for negative cases and the age at CRC diagnosis for positive cases. Body mass index (BMI) is likewise continuous. All such continuous factors were unitized to the interval [0, 1]. The sex factor is 0 for women and 1 for men. The variable of Hispanic ethnicity was given a value of 0 for a response of “Not Hispanic/Spanish origin” and 1 otherwise. The variable of race was set to 1 for responses of “Black/African American only,” “American Indian only,” “Other race,” or “Multiple race,” and 0 otherwise. The smoking status had a value of 1 for an everyday smoker, 0.66 for a some-day smoker, 0.33 for a former smoker, and 0 for a never smoker. The NHIS defines a “never smoker” as someone who has smoked 100 cigarettes or less over their entire lifetime, and a “former smoker” as a smoker who quit at least 6 months prior to the survey; this same definition was used to score PLCO respondents' smoking status using equivalent fields. The variable of family history represents the number of first-degree relatives who have had CRC, and was capped at 3. The family history variable values of 0, 1, 2, and 3 were mapped to 0, 0.33, 0.66, and 1, respectively.

### The Levels of TRIPOD and the Cross-Testing Uncertainty

Below, we use the terms “training,” “validation,” and “testing” to describe increasingly-general model performances. Any portion of data designated as “training” is used to directly adjust the parameters of the model (e.g., by iterations of gradient-descent in the space of model parameters for an artificial neural network). Any portion of data designated as “validation” is not involved in direct adjustment of model parameters, but is used to stop further iterations of an algorithm based on whether overfitting happens (e.g., stopping iterations of gradient descent if the training fitting error is decreasing but the validation fitting error is increasing). Finally, any portion of data designated as “testing” is data used for neither training nor validation. In the literature, the term “validation” is sometimes used to describe what is actually testing, often by way of the term “cross-validation” (Picard and Cook, 1984). In this work, we use the term “cross-testing” to avoid any possible confusion.

We reported concordance, a performance metric, at level 3 of the hierarchy proposed by the Transparent Reporting of Multivariable Prediction Model for Individual Prognosis or Diagnosis (TRIPOD) guidelines (Collins et al., 2015). TRIPOD level 1a corresponds to testing upon the same dataset used for training (leaving any overfitting undetected). TRIPOD level 1b corresponds to *n*-fold cross-validation (Picard and Cook, 1984). TRIPOD levels 2a and 2b each correspond to a trained model tested upon or cross-tested between splits of the data involved neither in training nor overfitting-detection (“validation”). Level 2a corresponds to random splits of the data, accordingly yielding normally-distributed random error (Bertsekas and Tsitsiklis, 2008) in Equation (2). Level 2b corresponds to non-random splits of the data, yielding systematically-distributed error in Equation (2). TRIPOD level 3 is where a model trained by data from one study is tested upon or cross-tested between data from a separate study. TRIPOD level 4 corresponds to testing a published model on a separate dataset (Appendix).

Our model has a TRIPOD level of 3, as it was trained upon a dataset from a longitudinal study and tested on a dataset from a cross-sectional study and vice versa. Throughout this paper, cross-testing shall refer to training on NHIS/PLCO and testing upon PLCO/NHIS, respectively. In this case, the systematic error from Equation (2) arises from the distributional disparity [in the Bayesian perspective (Bertsekas and Tsitsiklis, 2008), where each data entry in the NHIS and PLCO is assumed to be drawn from separate probability distributions with unknown parameters] between the PLCO and NHIS datasets due to (among other things) the fact that the NHIS is cross-sectional while PLCO is longitudinal. Reporting performance at TRIPOD level 3 demonstrates generalizability of the model's predictive capacity.

### Seven Machine Learning Algorithms

The MLAs used in this work are an artificial neural network (ANN), logistic regression (LR), naive Bayes (NB), decision tree (DT), random forest (RF), linear-kernel support-vector machine (SVM), and linear discriminant analysis (LDA) with automatic optimization of hyper-parameters (Fisher, 1936; Morgan and Sonquist, 1963; Rumelhart et al., 1986; Cortes and Vapnik, 1995; Hosmer and Lemeshow, 2000; Bertsekas and Tsitsiklis, 2008). The LR, NB, DT, SVM, and LDA MLAs were invoked, respectively, by the “fitglm,” “fitcnb,” “fitctree,” “fitcsvm,” and “fitcdiscr” MATLAB functions. The SVM, LDA, and DT MLAs yielded a CRC risk score using Platt scaling (Platt, 1999). The ANN used a previously developed in-house MATLAB code.

ANNs are a method of regression (Bertsekas and Tsitsiklis, 2008) as they determine function parameters that minimize fitting error using iterations of stochastic gradient descent in parameter space (Bishop, 2006; Andoni et al., 2014; Kingma and Ba, 2015) through a process called backpropagation (Rumelhart et al., 1986), and are similar to logistic regressions (LR) (Hosmer and Lemeshow, 2000). Specifically, an ANN with a logistic activation function and zero hidden layers is a logistic regression. With their hidden layers, ANNs model inter-factor coupling as logistic or hyperbolic-tangential probabilities; these probabilities are called the activation function of the ANN. ANNs that use logistic activation functions are multilinear generalizations of LRs.

The in-house MATLAB coded ANN has two hidden layers with logistic activation and deployed adaptive gradient descent via the “Adam” learning rate. It also uses both early stopping and automatic hyperparameter optimization to minimize overfitting. There is one input neuron for each factor used, and each hidden layer has one neuron for each input neuron. Each neuron is associated with a single weight *W* and a single bias *B*, which, respectively, are the slope and intercept for the linear function *z* = *z*(*X*) = *WX* + *B* with argument *X*. The linear function itself is then fed into the neuron's sigmoidal activation function (*e*^−*z*^ + 1)^−1^. The weights and biases are, respectively, determined by iterations of the equations $W^{\prime }=W-\alpha \frac{dS}{dW}$ and $B^{\prime }=B-\alpha \frac{dS}{dB}$ for fitting error $\text{S}=N^{-1}\ln\;\prod_{i=1}^{N}{{Ȳ}_{i}}^{Y_{i}}{\left(1-{Ȳ}_{i}\right)}^{1-Y_{i}}$ between the subject's risk-score and their actual cancer status in a total of *N* subjects, a process called backpropagation. In our backpropagation, we chose to iterate until |*W*′ − *W*|, |*B*′ − *B*| ≤ ε for a small ε we picked.

The NB method modeled the conditional probability of having CRC by constructing a Gaussian distribution with a conditional sample mean and conditional sample variance (Bertsekas and Tsitsiklis, 2008). This conditioning was based on whether or not each respondent was drawn from the CRC or the never-cancer population. That is, the conditional probability *P* = P(C|Φ) of the event Φ of having a set of features (e.g., hypertension, diabetes, body-mass index) resulting in the event C of having CRC was given by Bayes theorem as P(C|Φ) = P(Φ|C)P(C)/P(Φ). The NB method thus incorporated inter-factor coupling, though as a multiplicative model that assumed the factors to be distributed independently. Despite this assumption of independence being almost always incorrect, the NB method's performance was competitive with those of more advanced MLAs (Rish, 2001).

The LDA and SVM calculate a decision boundary between the positive and negative populations that maximized a likelihood function (Fisher, 1936; Cortes and Vapnik, 1995). The method assumed homoscedasticity, multicollinearity, and that the responses were random variables drawn from completely independent Gaussian distributions. The SVM method similarly calculated a decision boundary, except without assuming the feature-values were drawn from a Gaussian distribution. In general, decision boundary methods are effective because they resist the effects of outliers.

The DT method constructed a flowchart of factors leading to CRC. The DT used the variable of lowest entropy (Shannon, 1948; Morgan and Sonquist, 1963) to construct the base of the tree, and used increasingly less informative variables at higher branches. Such a flowchart can be easily understood by a human, and is thus highly desirable in a clinical setting. Finally, we tested a bootstrap-aggregated (“bagged”) collection of random trees, better known as a random forest (RF). Such RFs resist the overfitting that DT are prone to, but lack the transparency and information that DTs have in making their classifications.

### Six Imputation Methods to Handle Missing Data

The datasets were subject to mean, Gaussian, Lorentzian, one-hot encoding, expectation-maximization (EM), and listwise deletion to handle data that are missing completely at random (Little and Rubin, 2014), some of which over-represented distributional moments. The six examined imputation methods have different strengths and weaknesses. Imputation by mean over-represents the mean. Imputation by drawing from a Gaussian random variable over-represents the variance about the mean. Imputation by drawing from a Cauchy random variable does not over-represent the mean or variance. Imputation by one-hot encoding (Bishop, 2006) uses the actual missingness of a data-entry as a feature. Finally, imputation by the (multivariate Gaussian) expectation-maximization (EM) iterative method over-represents the covariance between features (e.g., the covariance of diabetes with hypertension). The methods that draw from the Gaussian and Cauchy distributions used MATLAB's random number generator in invoking the function “rand,” and thus are stochastic. Imputation by mean, one-hot encoding, EM algorithm, and listwise deletion, on the other hand, are deterministic.

The version of the EM algorithm that we chose assumed that all variables in each dataset were random variables drawn from a multivariate Gaussian distribution (Bishop, 2006). Iterations of the algorithm imputated the MCAR with values that overrepresented the covariance of each data column with each other. Since the multivariate Gaussian distribution is completely specified by its mean and variance, imputation by this method is incorrect only if the data is not drawn from a multivariate Gaussian or if the data is not MCAR. Because both the NHIS and PLCO datasets distinguish between data that is MCAR and data not missing at random, the effect of non-normal/Gaussian distribution of missingness remained minimized.

The multivariate-Gaussian EM algorithm is just one of many types of EM algorithms, as other data distributions (e.g., a multivariate-multinomial) may be assumed. Because our data contains a mixture of continuous and binary data-fields, and because the closed-form properties of the multivariate Gaussian are well-known (Bishop, 2006), we used Gaussian expectation-maximization for convenience. Categorical survey fields are multinomial, and a sufficiently-large number of such multinomial random variables are Gaussian by the central limit theorem (Bertsekas and Tsitsiklis, 2008). Ordinal survey fields have a distribution that in general have non-zero skewness and kurtosis, and thus are not exactly Gaussian. To avoid the calculation of the covariance of one multivariate Gaussian distribution with a non-Gaussian distribution, we just used a multivariate Gaussian for all fields. “Multivariate-Gaussian EM imputation” shall be referred to as just “EM imputation” throughout this paper.

About 1.2% of all data (795,215 respondents) was missing completely at random. However, about 16% of the 795,215 respondents had one or more of these missing entries. Listwise-deletion discards any respondent with even one missing entry, so about 16% of all data is then lost.

### Model Evaluation

A popular metric of the performance in discriminating CRC incidence from non-CRC incidence is concordance, which is sometimes known as the area under the curve (AUC) of the receiver-operator characteristic (ROC) plot (Hanley and McNeil, 1982; Hosmer and Lemeshow, 2000; Fawcett, 2005). We reported concordances from training on NHIS/PLCO and testing upon PLCO/NHIS (“cross-testing”), which gives a TRIPOD level (Collins et al., 2015) of 3. Total uncertainty in concordance across cross-testing (Picard and Cook, 1984) is calculated using Equation (3).

For individuals ages 18–49 the PLCO dataset^2^ has a sharply different prevalence of CRC (379 positives, 12 negatives) compared to the NHIS dataset^1^ (114 positives and 76,676 negatives for family history data used; 562 positives and 398,222 negatives for family history data not used). Thus, for this age range, models were cross-tested between the 2-folds formed by the following non-random split: (1) the combination of all PLCO data with NHIS years 1997–2006 and (2) the remaining NHIS years 2007–2017. This makes the testing level for individuals ages 18–49 drop from TRIPOD 3 to TRIPOD 2b.

### Stratifying CRC Risk

The ANN with EM imputation was used to stratify subjects into low-, medium-, and high CRC-risk groups. The ANN trained on NHIS data, and used this model to stratify the PLCO subjects into these risk categories. The PLCO dataset records the time in days at which the participant was diagnosed with CRC, and that time was used to build a forecast in the form of a Kaplan-Meier (KM) survival plot. Performance in risk-stratification was reported to give both an illustration of immediate clinical application and a performance metric that is not as biased as concordance is (Bertsekas and Tsitsiklis, 2008; Hajian-Tilaki, 2013).

## Results

### Concordance Statistics of Seven Machine Learning Algorithms

**Figure 1:**
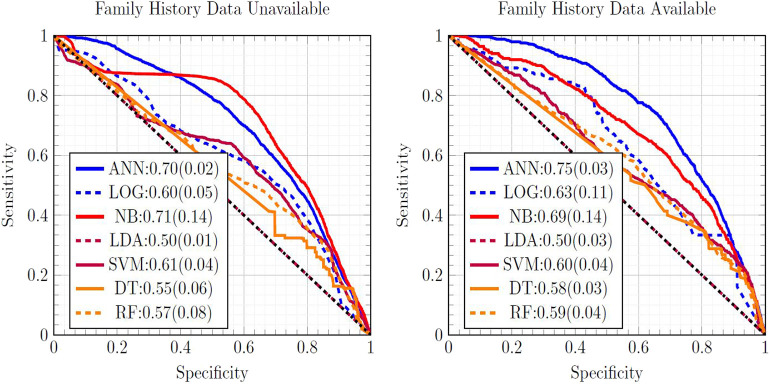
Comparison of ROC curves of all seven MLAs, with the mean concordance and its uncertainty reported. Expectation maximization was used to impute missing data.

Figure 1 is a ROC plot of the seven MLAs used with datasets subject to EM imputation. The standard deviation was formed from the variance from cross-testing between the NHIS and PLCO datasets, and the variance from screened/unscreened sub-populations (Hanley and McNeil, 1982) using Equation (3). Considering the mean concordance minus the total uncertainty (Equation 3) to be the metric of performance, the top performer was the ANN, with the SVM and NB as equally-performing runner-ups. LR (Hosmer and Lemeshow, 2000) offered fourth-place performance. Our ANN used the same logistic activation function (Bishop, 2006) as the LR. Our LR was our ANN with no hidden layers, suggesting the importance of inter-factor coupling possibly corresponding to complications. The good performance of the SVM came from not assuming a particular underlying distribution to the data, while LDA assumed that the NHIS and PLCO data were drawn from Gaussian distributions. The good performance of the NB came from its multiplicative incorporations of inter-factor coupling. The ANN's good performance was also roughly insensitive to which imputation method is used. The SVM and LDA perform well with one-hot encoding imputated data due to their resisting overfitting and outliers. RFs offered slightly improved performance over the DT, but worse than the ANN.

The concordance statistics for cross-testing for all combinations of MLAs and imputation methods are summarized in Table 1, showing relevant divisions of the datasets by age, as well as the effect of making family history data part of the model vs. leaving it out. The ANN offered performance (mean concordance minus the uncertainty) that was not only better than other MLAs but also insensitive to which imputation method was used. It can also be seen that in the group ages 18–49, among whom recent diagnosis of CRC is rarer (due in part to a greater prevalence of those bypassed for by-age screening of the USPSTF's recommendations), concordance was driven up by the increased true negative rate (or specificity). The opposite effect was observed in the group ages 50–75. Including family history data improved performance, but in a manner that is offset by the fact that it could only be included for a smaller data. Finally, it can be seen that the EM Gaussian algorithm tended to give the best concordance. One-hot encoding similarly performed well.

**Table 1:**
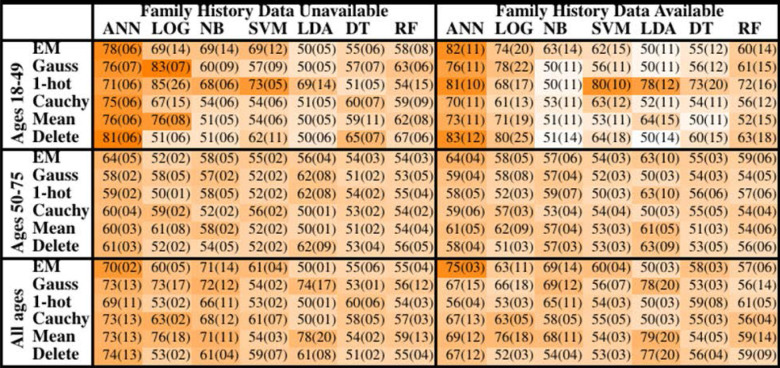
Mean concordance (standard deviation), multiplied by 100, for various machine learning algorithms, imputation methods, age groups, and with or without family history of CRC data.

*Models for all ages and ages 50–75 were conducted at TRIPOD level 3 and models for ages 18–49 at TRIPOD level 2b. The standard deviation reported has a component of population-uncertainty from Equation (1) and a cross-uncertainty from Equation (2). The cell shading scheme was determined by subtracting the standard deviation from the mean concordance statistic, so that darker shading indicates a concordance statistic that not only had a higher mean value, but also a lower uncertainty*.

### Testing the ANN at TRIPOD Level 3

**Figure 2:**
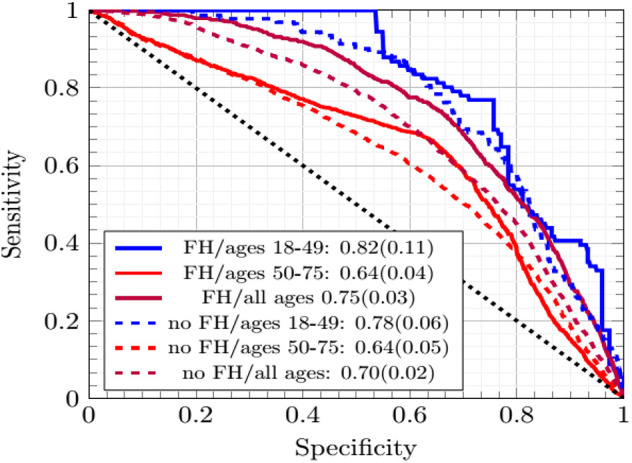
The ROC curves of the ANNs averaged across cross-testing for different sub-demographics and with/without family history data.

In Figure 2 the ANN with EM imputation performed consistently well as the incorporation of family history data and age range of subjects varied. The AUCs were greatest for individuals ages 18–49. The prevalence of CRC was lower in this group and Figure 2 shows that the concordance was driven up by the low-cutoff portion of the ROC curve where the sensitivity of the ANN with EM imputation can be seen to rise sharply. This sharp rise is due to the high probability of any negative call being correct in a dataset with such low prevalence of CRC. As the cutoff increases in Figure 2, the sensitivity exhibits several sharp drop-offs. In the high-cutoff portion of Figure 2, the performance of the ANN with EM imputation becomes insensitive to the age-demographic, or even the incorporation of family history data in the model. This trend is in sharp contrast to the low-cutoff portion of the ROC, where performance in the group of individuals ages 18–49 was significantly better than in the high-prevalence group of those ages 50–75. These results make the case that the concordance is a good measure of the performance of the ANN with EM imputation relative to other MLAs.

The improvement in concordance in Figure 2 and Table 1 is due to the ANN becoming increasingly insensitive when trained on data from individuals ages 18–49 and thus making more negative calls. The high number of negative calls in ages 18–49 gives a high specificity, and thus a high concordance.

### Risk Stratification by ANN at TRIPOD Level 3

**Figure 3:**
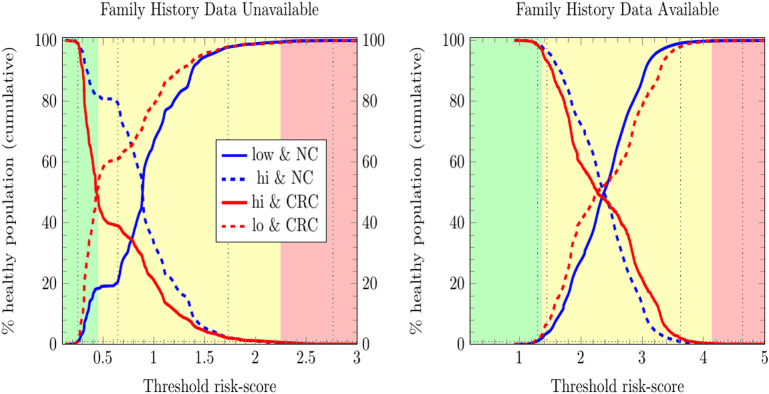
Stratification of individuals into low-, medium-, and high CRC-risk groups by the ANN with EM imputation. Risk categories are defined by the requirement that no more than 1% of positive cases be classified as low risk, and no more than 1% of negative cases be classified as high risk.

We stratified survey-respondents by the risk score calculated by the ANN with EM imputation. Such stratification has been completed at TRIPOD level 2b in previous work (Hart et al., 2018; Rofman et al., 2018), and was done here at TRIPOD level 3. Figure 3 illustrates the stratification of individuals into three risk score categories. Table 2 shows how many survey-respondents (CRC and never-cancer) ended up in each category.

**Table 2:**
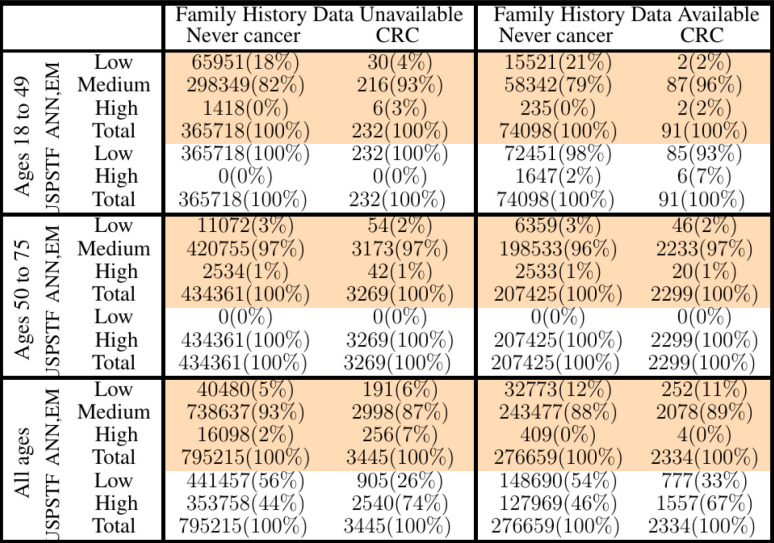
Comparison of our ANN with EM imputation with USPSTF screening guidelines in stratifying PLCO and NHIS respondents into low-, medium-, and high CRC-risk groups.

In Figure 3, only relative (rather than absolute) values of risk are relevant, and thus the numbering of the horizontal axis is not comparable between plots. Unitizing the axis of risk to the interval [0, 1] is not done. Doing so would be misleading because differing models have differing levels of risk because the minimum and maximum values of risk needed for unitization differ between models. In stratifying NHIS/PLCO using a PLCO/NHIS-trained ANN, the risk-boundaries constructed as such were in general not equal, and the interval formed by this disparity is demarcated by vertical black dotted lines. Cumulative functions and complement-cumulative functions of negative and positive populations are plotted. Dotted lines and solid lines of the same color are complementary cumulative distributions summing to 100%.

### Predicting CRC Incidence in the Never-Cancer Population

**Figure 4:**
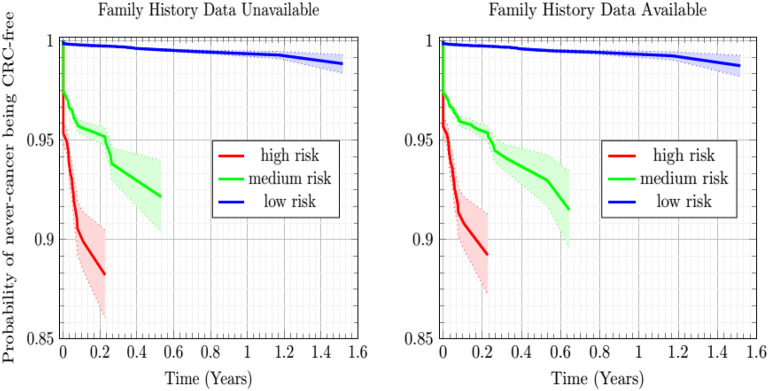
Plot of the Kaplan-Meier estimator of the CRC-free probability of the PLCO respondents vs. time for the low-, medium-, and high CRC-risk groups stratified by ANN with EM imputation model. The shaded regions are 95% confidence intervals at TRIPOD level 1a.

The Kaplan-Meier plots of Figure 4 show the estimated probability of the never-cancer PLCO population getting CRC as a function of time in years, taking the CRC population as a Bayesian given. A cone of uncertainty is indicated. This cone, which widens at later times, suggests that the never-cancer population flagged as high risk (see Figure 3 and Table 2) has an appreciable probability of developing CRC at a later time. Accordingly, this group regarded as “false positives” actually would benefit from screening. Because these false positives drive down the sensitivity and positive predictive value, this builds the case that concordance is better suited as a relative rather than absolute metric of performance.

In Figure 4, while the risk stratification into three categories is done at TRIPOD level 3, the confidence intervals are at TRIPOD level 1a. This is because they contain only a population-uncertainty calculated from an expression analogous to Equation (1).

## Discussion

### Machine Learning Algorithms for CRC Prediction

Obtaining a concordance of 0.70 ± 0.02 on training an ANN with EM imputation gives a test that is competitive with the tests using routine data itemized in the review by Usher-Smith et al. (2016), including Betes (Betes et al., 2003) (TRIPOD level 3) and even the self-completed questionnaire used by Colditz (Colditz et al., 2000) (TRIPOD level 3). Our model of CRC risk combines routine data (involving no biomarkers) to form a score of CRC risk, and thus gives a discriminating and generalizable score of CRC risk.

Like other clinical tests, the negative calls made by the ANN with EM imputation have a greater probability of being correct compared to the corresponding probability of correctness of its positive calls. This trend can be seen by considering the model's strong performance among individuals ages 18–49 as well as its sensitivity (0.63 ± 0.06) and misclassification rate of CRC as low risk being significantly worse than its specificity (0.82 ± 0.04) and misclassification rate of non-CRC as high risk. Among individuals ages 18–49, the concordance was driven up by the increase in specificity which was probably triggered by the greater number of respondents for whom the ANN with EM imputation could make correct negative calls. Likewise, better performance was observed when testing the PLCO-trained model upon NHIS data (compared to testing the NHIS-trained model upon PLCO data). In NHIS, there are ~10^6^ respondents of which ~10^3^ have CRC, whereas in PLCO there are ~10^5^ respondents of which ~10^3^ have CRC. Thus, for the PLCO dataset, there were (an order of magnitude) fewer specificity-boosting negative calls. In typical clinical practice such a test that makes a negative call gives a recommendation for no further testing, while a positive call gives a recommendation for further testing by a more accurate (and costly) test (Simundic, 2017).

This paper reported uncertainty at TRIPOD level 3 wherever possible. Reporting uncertainty is crucial to determine optimal performance because concordance can be misleadingly high even when averaged across cross-testing. For instance, imputation of missing data with the average of a data-field gave a concordance that was almost 0.80, but with an accompanying uncertainty of 0.20. The performance for which mean minus uncertainty was greatest was 0.70 ± 0.02 (see Table 1) when ANN with EM imputation were used.

### Improving Performance With Additional Relevant Factors

The input predictors to the MLAs were selected based on availability in both the NHIS and PLCO datasets, what Rubin calls the file-matching problem (Little and Rubin, 2014). Because of this selection criteria, some of the stronger factor-correlations with CRC (e.g., NSAIDs, such as aspirin and ibuprofen; Rodriguez and Huerta-Alvarez, 2001; Betes et al., 2003) needed to be omitted from the model, as data on use of NSAIDs was only available in the NHIS dataset for years 2000, 2005, 2010, and 2015. The risk-stratification demonstrated in Table 2 would likely be even more effective if these stronger predictors were used. Indeed, a data-driven approach to detecting CRC risk in the general public would put priority on recording these strong predictors more regularly.

### ANN With EM Imputation

The concordance of 0.70 ± 0.02 of our ANN with EM imputation is competitive with previous externally-tested (TRIPOD 3) risk models using routine data (Betes et al., 2003; Usher-Smith et al., 2016) as input. To our knowledge, calculating an uncertainty by the law of total variance (Bertsekas and Tsitsiklis, 2008) so as to incorporate both the population-uncertainty (Hanley and McNeil, 1982; Fawcett, 2005) of Equation (1) and the cross-uncertainty due to variance in performance across cross-testing (Picard and Cook, 1984) of Equation (2) has never been done before. Incorporating this additional component of cross-uncertainty demonstrates the advantage of using the ANN. The advantage of the ANN over LR is not in having a high mean concordance, but rather in having a much lower uncertainty, which demonstrates the generalizability of the model. Because of better generalizability, the ANN with EM imputation is considered the best among all the model/imputation configurations.

### Clinical Deployment

In this work, the developed ANN with EM imputation is used to predict the colorectal cancer risk for individuals based on their personal health data. The output of the model, the colorectal cancer risk score, can be used to help the clinicians make screening decisions. Generally speaking, true positives require further screening and true negatives require no screening. False positives still stand to benefit from our model, which offers this population their individual cancer risk as a function of personal health habits they have at different times. Drops in an individual's risk score in response to better personal health habits, such as quitting smoking and treatment of diabetes will provide positive feedback for that individual in the form of a reduced risk-score. Furthermore, high-risk never-cancer false positives warrant heightened screening attention, as demonstrated by the sharply decreasing Kaplan-Meier probability of high-risk never-cancer individuals remaining free of CRC over time. In general, the temporal trend of cancer risk will determine the next step for the individuals.

## Conclusion

In this comparative study, we have evaluated seven machine learning algorithms in combination with six imputation methods for missing data, all trained and cross-tested with the NHIS and PLCO datasets. Among various machine learning algorithms using different imputation methods, the artificial neural network with Gaussian expectation-maximization imputation was found to be optimal, with a concordance of 0.70 ± 0.02, a sensitivity of 0.63 ± 0.06, and a specificity of 0.82 ± 0.04. In CRC risk stratification this optimal model had a never-cancer misclassification rate of only 2%, and a CRC misclassification rate of only 6%. Being a TRIPOD level 3 study, our model with low uncertainty suggests that it can be used as a non-invasive and cost-effective tool to screen the CRC risk in large populations effectively using only personal health data.

## Data Availability Statement

The code used in this study is not publicly available due to a concern of intellectual property proprietary to Yale University. Requests to access the NHIS datasets should be directed to the Centers for Disease Control and Prevention (CDC) at https://www.cdc.gov/nchs/nhis/. Requests to access the PLCO datasets should be directed to the National Cancer Institute (NCI) at https://biometry.nci.nih.gov/cdas/plco/.

## Author Contributions

BN analyzed the data, produced the results, and wrote the technical details. GH, WM, YL, and GS produced the technical details, and reviewed the manuscript. JD generated the research ideas and reviewed the manuscript.

### Conflict of Interest

The authors declare that the research was conducted in the absence of any commercial or financial relationships that could be construed as a potential conflict of interest.

## Footnotes

^1^National Health Interview Survey (NHIS) (1997–2017).

^2^Prostate/Lung/Colorectal/Ovarian (PLCO) Cancer Screening Trial (1993–2001).

## Appendix

### The Levels of TRIPOD and the Cross-Testing Uncertainty

Differing levels of TRIPOD have cross-testing uncertainty from different sources of distributional disparity. There is always a component of uncertainty due to the finitude of the dataset used, and that of the concordance is well-known (Hanley and McNeil, 1982; Fawcett, 2005). For a population of N respondents C of whom have cancer and a MLA giving a concordance of AUC, what shall be called the “population-uncertainty” Π^2^ is a function of AUC, C, and N alone and given as,

(1) $$\begin{array}{l} \Pi^{2}=\frac{1}{C(N-C)}\left(\begin{array}{l} AUC\left(1-AUC\right)+\left(C-1\right)\left(\frac{AUC}{2-AUC}-AUC^{2}\right)\\ \;+\left(N-C-1\right)\left(\frac{2AUC^{2}}{1+AUC}-AUC^{2}\right) \end{array}\right) \end{array}$$

For TRIPOD level 1b or higher, an additional uncertainty component we call the “cross-uncertainty” (τ^2^) arises from cross-validation or cross-testing. This uncertainty, unlike the population-uncertainty, depends explicitly upon the disparity of the distribution of the two datasets. If the split of the data is random (TRIPOD levels 1b and 2a), the cross-uncertainty is normal or Gaussian (Bishop, 2006; Bertsekas and Tsitsiklis, 2008). If the split of the data is non-random (TRIPOD levels 2b and 3), the cross-uncertainty indicates the difference between the distributions of the data of each group. In the case of cross-testing between NHIS and PLCO, the cross-uncertainty indicates the difference between the underlying distributions of each dataset. For splitting of the data into *n*_f_-folds the cross-uncertainty τ^2^ is estimated as the sample variance resulting from the concordance AUC_i_ from testing or validating upon the ith-fold of data summed over all *n*_f_-folds, which is done as:

(2) $$\begin{array}{l} \tau^{2}=\frac{1}{n_{f}-1}\overset{n_{f}}{\underset{i=1}{\sum }}{\left(AUC_{i}-\bar{AUC}\right)}^{2};\;\bar{AUC}=\frac{1}{n_{f}}\overset{n_{f}}{\underset{i=1}{\sum }}AUC_{i}; \end{array}$$

A true positive rate or sensitivity (TPR) and a true negative rate or specificity (SPC) determine a concordance or area under the (receiver-operator characteristic) curve AUC. Taking TPR and SPC to be random variables that take on differing values over differing folds of cross-testing, we used the law of total variance (Hosmer and Lemeshow, 2000) to form the uncertainty σ^2^. Both the population-uncertainty Π^2^ and the cross-uncertainty τ^2^ between folds of data were incorporated as the following sum of variances conditioned upon a specific TPR and SPC:

(3) $$\begin{array}{l} \sigma^{2}=E\left[\text{var }AUC|TPR,SPC\right]+\text{var }E\left[AUC|TPR,SPC\right]\\ \;=\frac{1}{n_{f}}\overset{n_{f}}{\underset{i=1}{\sum }}\Pi_{i}^{2}+\tau^{2}=\bar{\Pi_{i}^{2}}+\tau^{2} \end{array}$$

Throughout this paper, we reported the square root of the total uncertainty $\sqrt{\sigma^{2}}=\sigma >0$ from Equation (3), formed from summing the mean population-uncertainty $\bar{\Pi_{i}^{2}}$ from Equation (1) and the cross-uncertainty τ^2^ from Equation (2). Through the cross-uncertainty τ^2^ the disparity between the distributions of the folds of cross-testing or cross-validation appear explicitly in the total uncertainty σ^2^.
